# Cationic Niosomes as Non-Viral Vehicles for Nucleic Acids: Challenges and Opportunities in Gene Delivery

**DOI:** 10.3390/pharmaceutics11020050

**Published:** 2019-01-22

**Authors:** Santiago Grijalvo, Gustavo Puras, Jon Zárate, Myriam Sainz-Ramos, Nuseibah A. L. Qtaish, Tania López, Mohamed Mashal, Noha Attia, David Díaz Díaz, Ramon Pons, Eduardo Fernández, José Luis Pedraz, Ramon Eritja

**Affiliations:** 1 Institute for Advanced Chemistry of Catalonia (IQAC-CSIC), Jordi Girona 18-26, E-08034 Barcelona, Spain; sgrgma@cid.csic.es (S.G.); ramon.pons@iqac.csic.es (R.P.); 2 Networking Center on Bioengineering, Biomaterials and Nanomedicine (CIBER-BBN), E-08034 Barcelona, E-01006 Vitoria-Gasteiz and E-03202 Elche, Spain; gustavo.puras@ehu.eus (G.P.); jon.zarate@ehu.eus (J.Z.); miriam.sainz@ehu.eus (M.S.-R.); nusaiba.qtaish@gmail.com (N.A.L.Q.); tblopez01@gmail.com (T.L.); e.fernandez@umh.es (E.F.); joseluis.pedraz@ehu.eus (J.L.P.); 3 NanoBioCel Group, Laboratory of Pharmaceutics, School of Pharmacy, University of the Basque Country (UPV/EHU), Paseo de la Universidad 7, 01006 Vitoria-Gasteiz, Spain; mahmed001@ikasle.ehu.es (M.M.); noha.attia@alexmed.edu.eg (N.A.); 4 Instituto de Productos Naturales y Agrobiología del CSIC, Avda. Astrofísico Francisco Sánchez 3, 38206 La Laguna, Tenerife, Spain; d.diaz.diaz@ipna.csic.es; 5 Institut für Organische Chemie, Universität Regensburg, Universitätsstr. 31, 93053 Regensburg, Germany; 6 Neuroprothesis and Neuroengineering Research Group, Miguel Hernández University, E-03202 Elche, Spain

**Keywords:** antisense oligonucleotides, aptamers, cationic lipids, cationic niosomes, gene delivery, plasmids, small interference RNA, therapy

## Abstract

Cationic niosomes have become important non-viral vehicles for transporting a good number of small drug molecules and macromolecules. Growing interest shown by these colloidal nanoparticles in therapy is determined by their structural similarities to liposomes. Cationic niosomes are usually obtained from the self-assembly of non-ionic surfactant molecules. This process can be governed not only by the nature of such surfactants but also by others factors like the presence of additives, formulation preparation and properties of the encapsulated hydrophobic or hydrophilic molecules. This review is aimed at providing recent information for using cationic niosomes for gene delivery purposes with particular emphasis on improving the transportation of antisense oligonucleotides (ASOs), small interference RNAs (siRNAs), aptamers and plasmids (pDNA).

## 1. Introduction

Nucleic acids drugs are becoming essential therapeutic molecules for precise medicine. Firstly, gene therapy has demonstrated that the deficiency of a protein can be overcome by the delivery of DNA vectors (usually plasmid DNA, pDNA) carrying the absent gene [1]. Secondly, the use of synthetic oligonucleotides for silencing overexpressed proteins has been validated using several approaches. The antisense approach demonstrated that single-stranded DNA complementary to mRNA could be used to block the translation of specific mRNA [2]. This technology generated the first oligonucleotide drugs available on the market [3]. Later, this approach was used to target exon sequences and modulate RNA splicing. Advances in the exon-skipping approach have resulted in successful treatments of Duchene muscular dystrophy and spinal muscular atrophy [2,3]. The discovery of RNA interference (RNAi) and microRNAs (miRNAs) has triggered intense research activity in the development of modified RNA as drugs [4]. Short interfering double-stranded RNAs (siRNAs) have shown themselves to be efficient for gene downregulation of a selected mRNA which tends to degrade through the RNA-interfering silencing complex (RISC). The first siRNA for human use was approved by the Food and Drug Administration (FDA) in 2018 [5]. In addition, nucleic acids can also be used to block or interact with proteins. Specific nucleic acids sequences such as aptamers [6] have been developed by screening combinatorial nucleic acid sequence libraries (Systematic Evolution of Ligands by EXponential enrichment, SELEX). Nowadays, there is no doubt that nucleic acids can be used to interfere with the cellular metabolism in a way that can generate novel medicines with more specificity and less toxicity than classic small drugs [3].

However, nucleic acids still have some drawbacks for their development as therapeutic agents. Chemical modifications of nucleic acids have been shown to improve their properties in terms of stability against nucleases and decreasing their off-targets effects, without losing their biological activities [4,7]. Nevertheless, the delivery issue is still one of the most important problems in the development of nucleic acids as drugs [8]. Although viral vectors like retroviruses or adenoviruses have shown high transfection efficiencies and been used in clinical trials [9] certain concerns regarding immunogenicity or recombination of oncogenes must be overcome. In contrast, non-viral vectors such as lipids [10], cell-penetrating peptides [11], polymers [12] or gold nanoparticles [13] have emerged as promising alternatives in order to deliver nucleic acids safely. Although several advances have been described in the search for formulations for nucleic acids, there is still a need for novel and more efficient delivery systems.

Liposomes constituted by phospholipids [14,15], lipidoid or lipid nanoparticles [16,17], cationic lipids [18] and cationic polymers [12,19] are the most frequently used non-viral vectors for nucleic acids transfection. From these alternatives, liposomes and other lipid formulations are most popular in human treatment. It has been demonstrated that of the majority these lipid formulations accumulate in the liver through the intervention of apolipoproteins [20]. On the other hand, niosomes are considered attractive alternatives for the generation of new formulations for gene and oligonucleotide transfection. Niosomes are non-viral vectors similar to liposomes in which phospholipids have been replaced by non-ionic surfactants [21,22]. In addition to non-ionic surfactants, niosomes for gene delivery contain one or more cationic lipids so nucleic acids are complexed to cationic niosomes through simple electrostatic interactions [23]. The differences in the chemical composition between liposomes and niosomes allow for the preparation of lipid formulation at a lower cost, longer stability and less toxicity. For a general overview of niosomes, preparation methods, drug administration routes and applications in the delivery of small molecules, two excellent articles have been published [24,25]. In this review, we aim to describe recent results in the use of cationic niosomes as alternative formulations for gene and oligonucleotide delivery.

## 2. Composition, Preparation and Characterization of Niosomes

### 2.1. Composition of Niosomes

**Figure 1:**
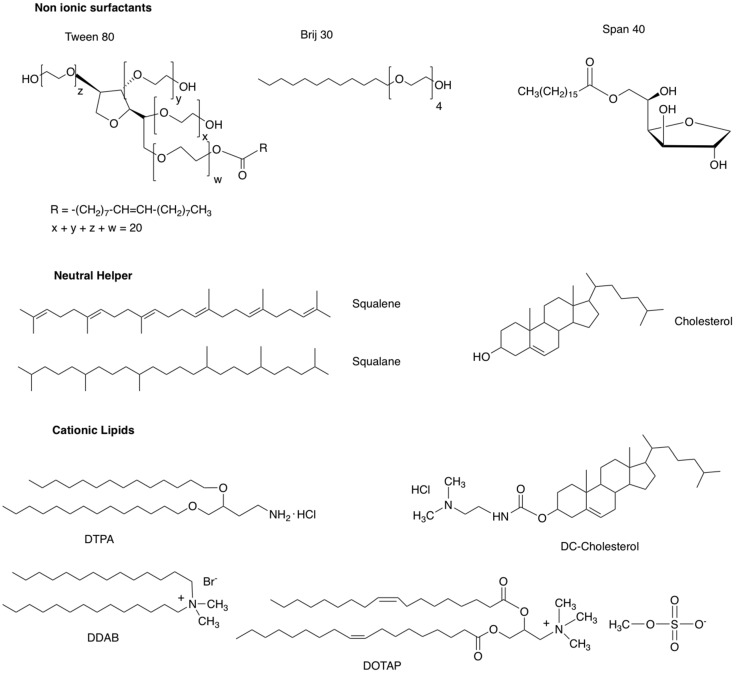
Chemical structure of some of compounds used in the composition of niosomes.

Cationic niosomes for nucleic acid delivery are made up of several elements including: (1) a non-ionic surfactant such as polyoxyethylene fatty acid esters or polysorbates (Tween^®^ [26]), polyoxyethylene alkyl ethers (Brij^®^ [27]), sorbitan fatty acid esters (Span^®^ [28]); (2) a neutral helper lipid such as cholesterol or squalene (SQ) or squalene (SQL) [29,30]; and (3) a cationic lipid such 3β-[*N*-(dimethylaminoethane)-carbamoyl]-cholesterol hydrochloride salt (DC-Chol, [31]), *N*-[1-(2,3-Dioleoyloxy)propyl]-*N*,*N*,*N*-trimethylammonium methylsulfate salt (DOTAP, [32]), dimethyldidodecylammonium bromide (DDAB, [33]) or 2,3-di(tetradecyloxy)propan-1-amine hydrochloride salt (DTPA, [34]) (Figure 1).

The relative amount of the different components is adjusted during the development of the formulations. Non-ionic surfactants are responsible for stability and lack of toxicity since they do not have charges. Helper lipids are neutral lipids that increase colloidal stability but also have an impact on cellular uptake and the internalization mechanisms of niosomes [30]. Finally, cationic lipids are needed to form electrostatic complexes with the negatively charged nucleic acids. A careful balance of the final charge of the nioplexes is needed to avoid aggregation or rapid removal from circulation by the reticuloendothelial system (RES) [21].

Other additives may also be present in niosomal formulations used for nucleic acid transfection. These include polyethyleneglycol (PEG) derivatives to prevent premature removal from circulation by the RES [35], protamine to increase DNA condensation and transfection rates [36] and poloxamers to enhance cellular uptake and viability in glial cells [37].

### 2.2. Niosome and Nioplexes Preparation

Several methods have been described for the preparation of niosomes [23,38,39]. In the solvent emulsion-evaporation technique, the helper lipid and the cationic lipid are dissolved in a volatile organic solvent such as dichloromethane and the non-ionic surfactant is dissolved in water. Mixing both solutions gives rise to a mixture of two phases that is emulsified with a sonicator. Thus, the organic solvent evaporates while dispersion is magnetically stirred and the emulsion is separated from the aqueous solution.

In the thin film-hydration method, the helper lipid and the cationic lipid are dissolved in a volatile organic solvent and the solution is evaporated in a large flask in order to obtain a lipid film that is mixed with a non-ionic surfactant molecule which is ultimately hydrated in the presence of water with the aid of a sonicator. Alternatively, the evaporation of the volatile solvent may be carried out by gently mixing it with a magnetic stirrer and the hydration of the film with the aqueous solution of the non-ionic surfactant can carried out by gently mixing it by shaking it with your hand. In the solvent injection method, both the lipid solution of a helper lipid and the cationic lipid in an organic solvent are injected into the aqueous solution of the non-ionic surfactant. An alternative for preparing niosomes without the use of an organic phase is the “bubble” method. In a glass reactor with three necks, the components of niosomes are dispersed at a high temperature. The first neck of the glass reactor is used to control the temperature with a thermometer. The second neck is used to supply nitrogen flow and the third one is used for the water-cooled reflux. Such dispersion containing the niosome components is mixed for a short period of time with a high shear homogenizer and is followed by bubbling with nitrogen [27].

All these above mentioned methods are simple but require high shear homogenization, ultrasounds or magnetic stirring. In these conditions it is difficult to control the size and homogeneity of the niosomes. However, this issue can be solved by using continuous-flow microfluidic methods. In this approach, the lipid solution of the helper lipid and the cationic lipid in organic solvent is mixed with the aqueous solution of the non-ionic surfactant by using a microfluidic system that renders a more homogeneous and reproducible niosome solution [40,41].

Once the niosomes are prepared using any of the techniques mentioned above, they can be stored at 4 °C for several weeks without affecting the main physicochemical parameters involved in nucleic acid delivery. Nioplexes can be easily obtained by electrostatic interactions after the complexation of a solution containing nucleic acids on the surface of cationic niosomes. The relative amount of nucleic acids and niosomes will depend on the nature of the nucleic acid. Normally, nucleic acids and niosomes are mixed in different proportions in order to obtain corresponding nioplexes, which leads to stock formulations that usually contain around 1 mg cationic lipid per mL and approximately 0.5–1 mg of plasmid DNA per mL [23].

### 2.3. Characterization of Niosomes and Nioplexes

One of the most important parameters related to the final performance of colloidal dispersions is size distribution. Normally, Dynamic Light Scattering (DLS) is used to determine hydrodynamic diameter and the polydispersity index (PDI) of both niosomes and nioplexes. Such parameters can be measured using a Zetasizer instrument. The same instrument can also be used to measure the zeta potential (ζ-potential) which is a critical parameter related to the charge of both niosomes and nioplexes formulations and therefore it is intimately related to the stability of these colloidal dispersions. In addition, the size and size distribution of formulations can also be measured using transmission electron microscopy (TEM) or even better, with cryo-TEM. However, these techniques are more difficult to use as they require staining the sample. In addition, the instruments are not usually available in many laboratories. However, if they are available, they can provide not only size and size distribution data but also morphology of the niosome and/or nioplexes.

Another important parameter is the stability of the formulations which can be measured by repeated analysis of their size, PDI and ζ-potential over a period of time at different temperatures. The complexation of niosomes with nucleic acids is usually determined by electrophoretic shift using gel electrophoresis. Plasmid DNA and oligonucleotide complexes are usually analysed using agarose and polyacrylamide gels, respectively. In both cases, the formation of the complexes is observed by retardation of the nucleic acid band that stays near the pocket instead of moving along the gel, which indicates condensation of the genetic material on the surface of the niosomes. In the case of the plasmid DNA, the electrophoretic analysis also allows us to determine DNA protection against enzymatic digestion mediated by nucleases along with the release of the nucleic acid cargo after adding anionic surfactants like sodium dodecyl sulphate (SDS) [29]. In this sense, the delicate balance between the condensation of DNA and its release needs to be achieved in order to enhance transfection efficiency of non-viral vectors based on cationic nanoparticles [42]. Additionally, analysis of the interactions between the cationic lipids and the genetic material at the molecular level can be measured using isothermal titration calorimetry. Such an analysis can provide relevant information about the physical mechanisms involved in the formation of nioplexes and their macroscopic and microscopic structure which are related to their final performance as nucleic acids delivery systems [43].

Another parameter that was used in the evaluation of niosome formulations is the pK_a_ value of niosome formulations, a parameter that may predict the delivery efficacy of these formulations [16]. There are two different methods for measuring amino lipid pK_a_ values: (1) potentiometric titration of amino lipids prepared in niosomes and (2) the effect of 2-(*p*-toluidino)-6-naphthalene sulfonic acid (TNS) fluorescence at different pH values containing ionizable amino lipids [44]. This second approach is usually the more convenient way for measuring the pK_a_ of cationic lipids in niosome formulations [45,46]. It has been suggested that the optimal pK_a_ of cationic lipids for transfection should be <7.0. In in this way, a large number of amino groups are neutral at physiological pH 7.4 [16].

### 2.4. Small Angle X-ray Scattering (SAXS)

Small angle X-ray scattering (SAXS) analysis of cationic lipids used in niosome preparation was used to determine the characteristics of the bilayers made up of several cationic lipids with different cationic groups [47,48,49,50].

Characterization of bilayers or multilayers is of utmost importance in understanding their behaviour and to be able to tailor their uses. SAXS (and also the equivalent technique of Energy Dispersive X-Ray Diffraction, EDXD) can provide information on bilayer thickness, hydrophilic and hydrophobic domain sizes as well as on hydration [46,47,51]. In multi-layered systems, additional information about correlated bilayers numbers and bilayer elasticity can also be obtained.

In addition, hints about bilayer asymmetry can be obtained using appropriate models and making several assumptions [50,52]. While dealing with phospholipid and biomimetic bilayers in great detail may require both high quality data, the combination with Small Angle Neutron Scattering (SANS) may result in simpler bilayers and gross modification of symmetry. This process occurs by adsorption or asymmetrical incorporation of additives and therefore results are more easily interpretable [50]. The electronic density profile across the membrane, the unambiguous characterization of the system as interdigitated or non-interdigitated are related to the thickness of the hydrophobic part of the bilayer. Furthermore, this is related with the easiness of pore formation and permeability which is also associated with the fluidity of the membrane. The extent of hydration of the polar heads is related with the colloidal stability of the vesicles and also with the interactions with biological materials. The location of additives is related with the encapsulation capacity and release.

## 3. Applications of Niosomes in Gene Delivery

One of the first applications of niosomes was reported by the cosmetic industry in the seventies [53]. Because of their structural similarities to liposomes and other colloidal systems, niosomes were also proposed as potential vehicles for transporting all sorts of drug molecules. Such features have confirmed the feasibility of niosomes as depots to facilitate targeted drug delivery by promoting sustainable release of potential small drugs in specific cells and tissues. In addition, niosomes have proved to be stable particles which have allowed to give enhanced stabilities to a good number of encapsulated drugs [54]. There are different routes that have been used to administer niosomal drugs like oral (e.g., Methotrexatetopical, Flurbiprofen); ocular (e.g., Chloramphenicol, Acetazolamide, Fluconazole) or topical administrations (e.g., Erythromycin, Minoxidil, Rofecoxib, among others) [55]. Furthermore, the effectivity and safety of such routes have been confirmed in numerous preclinical and clinical studies which have put an emphasis on biocompatible, biodegradable and low immunogenicity features of their components (non-ionic surfactants, cholesterol and other fatty acids as well as charged molecules).

Toxicity is also a factor that should not be overlooked when niosomes tend to be used in therapy. It is well known that cationic lipids have shown certain toxicity in transfection due to the presence of cationic charged molecules reducing their therapeutic effect. Thus, the presence of non-ionic molecules in niosomes might contribute to reducing such undesirable toxicity showing better cellular viability profiles than their corresponding anionic or cationic counterparts [27]. Therefore, all these properties as well as their low production cost and ease of preparation, have allowed niosome formulations to demonstrate multiple advantages over liposomes and other conventional drug delivery systems [56]. In fact, pharmaceutical companies have taken advantage of this niosome adaptability by using it in multiple applications (e.g., nutraceuticals, anti-bacterial, anti-oxidant and anti-cancer, among others) [27].

Gene therapy has proved to be an efficient approach for treating inherited human diseases when non-viral vehicles are involved in improving the cellular uptake properties of nucleic acids [50,57,58,59]. In particular, this review article is devoted to highlighting and discussing recent innovations and the development of niosome-based gene therapy strategy to overcome the transport of plasmids, antisense oligonucleotides (ASOs), small interference RNAs (siRNAs) and aptamers in target cells.

### Plasmids

Transfection of plasmids is an important tool in therapy in order to express a particular protein that is lacking in certain human cells [60]. Several strategies have been carried out in order to safely administer plasmid DNA in vivo in either physical methods like direct injection, systemic injection, electroporation and ultrasound techniques or using chemical carriers like cationic lipids, peptides and polymers, among others [61].

Cationic lipids are of extraordinary interest for great ability to interact with DNA plasmids and other genetic materials. Although some cationic materials are somewhat toxic and that limits their use in clinical studies, novel designs and subsequent optimization in structure-transfection activity relationships (SAR) have improved their safety and biodegradability properties [62,63]. Cationic lipids are made up of three defined parts: i. A hydrophobic tail based on saturated or unsaturated hydrocarbonated alkyl chains; ii. A positively charged head group that is responsible for promoting the electrostatic interaction between lipids and genetic materials and iii. a backbone that covalently connects the hydrophobic part with polar-head groups. Since the pioneering studies carried out by Felgner [64], a large number of lipids containing quaternary ammonium pendent groups [e.g., DOTMA, DOTAP or DORIE), polyamines (e.g., DOGS (dioctadecylamidoglycylspermine) or DOSPA (1,2-di-*O*-oleyloxysperminecarboxamidoethyl-dimethypropanaminium)]) and guanidines [i.e., BGTC (bis-guanidinium-tris(2-aminoethyl)-amine-cholesterol)] have been prepared and their transfection potency has been evaluated [65,66,67,68].

**Figure 2:**
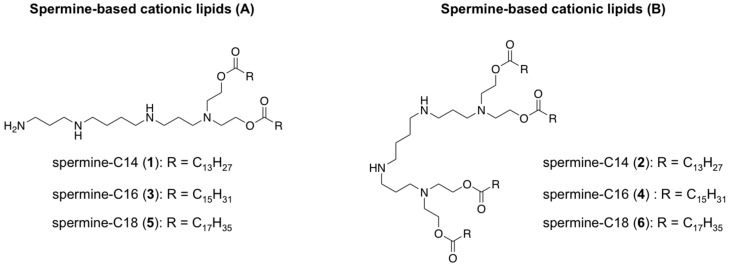
Spermine-based cationic lipids synthesized and evaluated as non-viral carriers for DNA plasmid.

Polyamines have proved to be efficient vehicles for drug delivery. This has sparked growth in the synthesis of novel polyamine derivatives [68] and bringing them into various niosome formulations. Several families of spermine-based cationic lipids have been designed which has led to potent and efficient non-viral carriers for DNA plasmids. In particular, the spermine molecule has been chemically modified by varying the chain length with symmetrical hydrophobic alkyl residues. Such hydrocarbonated fatty acids have modified both at the one and the two termini of the spermine building block producing the following diester families: spermine-C14 (**1**, **2**), spermine-C16 (**3**, **4**) and spermine-C18 (**5**, **6**) (Figure 2). These derivatives were formulated into niosomes using a thin-film hydration method in the presence of cholesterol (Chol) and Span-20 as a non-ionic surfactant molecule in a molar ratio of 1:2.5:2.5. These prepared niosome formulations were prone to interacting electrostatically with DNA plasmid encoding green fluorescent protein (pEGFP-C2) at different weight ratios. Interestingly, the behaviour of both spermine families was different in terms of particle size, complex formation and transfection efficiency. Thus, particle sizes in the first spermine family (A) ranged from 200 to 500 nm (213, 315 and 487 nm for **1**, **3** and **5**, respectively) while in the second family, (B) sizes were slightly bigger in most cases (876, 462 and 385 nm for **2**, **4** and **6**, respectively) (Table 1). As expected, agarose gel electrophoresis showed different weight ratio values when DNA was condensed into the niosome formulations. After confirming that DNA complexes did not affect cellular proliferation and did not have a haemolytic effect, the in vitro transfection efficiency of each formulation was evaluated with their optimal weight ratio. Gene transfection mediated by the two families of spermine-based cationic lipids also demonstrated different behaviour. While the niosome formulation **1** showed the highest transfection efficiency and this decreased as the hydrophobic alkyl chain increased, this tendency surprisingly changed in the case of spermine family B. Herein niosome formulation **6** was able to promote gene transfer more efficiently than the rest of the derivatives (Table 1). This different response was attributed to the ability of spermine-based cationic lipids **1** and **6** to be protonated at acid pH resulting in destabilization of the endosomal membrane [69,70].

**Figure 3:**
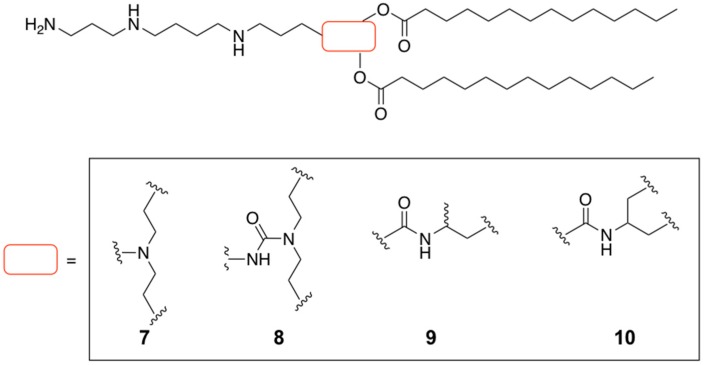
Design of a small library of spermine-based cationic lipids modifying the central core.

As part of a program focused on developing novel cationic lipids based on spermine analogues addressed to improve the delivery of DNA plasmid, the same authors were able to introduce the same fatty acid residue (C14) as described on Figure 2 but by varying the chemical structure of the spacer. Four more analogues were proposed (di(oxyethyl)amino (**7**), di(oxyethyl)aminocarboxy (**8**), 3-amino-1,2-dioxypropyl (**9**) and 2-amino-1,3-dioxypropyl (**10**)) [71] (Figure 3). These four spermine analogues were formulated into the same niosomes (Chol and Span-20) as described above. The authors carried out exhaustive physical characterization of the particle size by varying the amount of cationic lipid. As a consequence, the corresponding DNA particles had sizes that ranged from 94 to 195 nm, remarkably smaller than those shown above [14,15]. Before evaluating the efficiency of these cationic formulations with DNA plasmid (pEGFP-C2), exhaustive cytotoxicity and serum stability studies were also carried out. The authors found it had minimal effect on cellular viability and significant stabilities of the particles for up to 6 h. In vitro transfection experiments were carried out using the selected cationic lipids with a molar ratio of 0.5. This resulted in an optimized spermine-based formulation containing Span20:Chol:**8** (2.5:2.5:0.5) which shown promising ability to impart cellular uptake in HeLa cells.

Recently, this study allowed the same authors to select the spermine cationic lipid **8**, as a basic proof-of-concept for the delivery of a particular cargo. Specifically, this optimized formulation was used to transfect plasmid DNA-encoding ovalbumin (pOVA) by obtaining the corresponding cationic lipid **8**:pOVA particles [72]. The authors noticed that the optimized formulation in combination with a hollow microneedle device produced a sharp therapeutic action in vivo without observing any infections on the skin. Therefore, the strategy involving the transfection of pOVA complexes induced a good number of positive responses like interleukin-4, immunoglobulin and interferon gamma when compared to the effect produced by naked pOVA. This novel strategy has been recommended as an interesting method for vaccination.

Other cationic lipids have been proposed as an alternative to spermine derivatives. Manosroi et al. proposed to formulate dimethyldioctadecylammonium bromide (DODAB) into elastic niosome formulations. The efficiency of this lipid was previously studied in a good number of cell lines leading to transfections with close to 95% efficiency [73]. The strategy followed by the authors was based on evaluating the expression of the human tyrosinase gene (pMEL34) by promoting transdermal absorptions in order to find a reliable topical strategy for treating depigmented skin [74]. Following the well-known film hydration method, the authors were able to load pMEL34 in a mixture made up of Tween-61:Chol:DDAB (1:1:0.5 molar ratio). This protocol obtained maximum encapsulation values of 150 µg in 16 mg of the formulation showing ζ-potential values of 32.1 mV and particle sizes of 689 nm for this optimal mass ratio. Furthermore, elastic niosomes displayed higher thermal stabilities for up to 5 weeks when measured at different temperatures (4, 27 and 45 °C) if compared to non-elastic ones since the cargo precipitated after two weeks.

To understand this behaviour, the authors have suggested that the presence of ethanol might act as a hydroxyl scavenger and therefore protects the corresponding cargo from precipitation. However, these elastic niosomes were only found to be more stable at lower temperatures because of ethanol evaporation [75]. The authors found an optimal therapeutic response when pMEL34 was loaded in elastic niosomes after using Franz diffusion cells. As a consequence, this approach gave rise to the most intense tyrosinase activity (0.36 ± 0.03 U/µL), approximately four times greater when compared to non-elastic niosomes and non-loaded plasmid thus showing the potential use of elastic niosomes in transdermal delivery.

**Figure 4:**
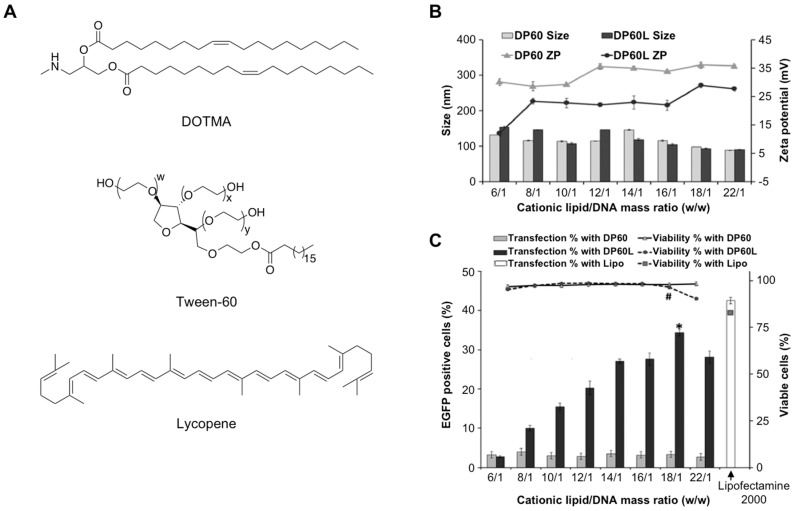
Particle characterization and in vitro gene transfection experiments mediated by niosomes made up DOTMA, Tween-60 and Lycopene. (**A**) Chemical structures; (**B**) particle size and zeta potential of nioplexes; (**C**) cellular viabilities and gene transfection after 72-h incubation in ARPE-19 cells. Control positive was carried out with lipofectamine. Adapted with permission from ref. [78]. Copyright 2017, Elsevier B.V.

Retinal gene therapy has become a promising therapeutic approach to treating disease-causing inherited ophthalmic pathologies [76]. While using viral vectors have proved to be more efficient than non-viral vehicles, they may face certain limitations in terms of safety, carcinogenesis, production and DNA packaging content, among others [77]. To overcome all these limitations, cationic niosomes have emerged as interesting nanovehicles in this types of diseases. Recently, Mashal et al. prepared a cationic formulation based on mixing Tween-60:DOTMA:lycopene according to the reverse-phase evaporation method (Figure 4A) [78]. This approach allowed these authors to study the effect of lycopene(a natural lipid carotenoid [79] with excellent antioxidant properties), not only on the stability of the niosomal formulation but also on transfection efficiency as it may act as a “helper” lipid.

The physical characterization showed that the particle size of niosome formulations increased when lycopene was introduced (66.4 vs. 101.60 nm, respectively) whereas the ζ-potential value was slightly lower (45.3 vs. 33.8 mV, respectively) (Figure 4B). DNA plasmid (pCMS-EGFP) was able to condense and form the expected nioplexes showing nanometric size and positive ζ-potential at the optimal mass ratio (18:1; cationic lipid:DNA). In vitro transfection experiments in ARPE-19 cells showed approximately 35% positive cells without affecting cellular viabilities. Therefore, the presence of lycopene in the niosome-based formulation exceeded transfection efficiencies when compared to their counterparts. Although this value was slightly lower when gene transfection was carried out using lipofectamine (42.6%), this new formulation might be a reliable option for ocular gene therapies (Figure 4C).

Further internalization studies confirmed that nioplexes containing the helper lipid followed caveolae and macropinoncytosis-mediated endocytosis processes. Finally, the authors confirmed the ability of this formulation to transfect the retinal outer segments in vivo using subretinal and intravitreal injections at the optimal mass ratio (18:1). Interestingly, the same niosome-based formulation based on DOTMA and lycopene as a cationic lipid and helper lipid, respectively was studied for delivering genetic material into the brain. Transfection experiments displayed moderate efficiencies in NT2 cells (17%) at mass ratio of 14:1 following clathrin and caveolae as the predominant endocytosis pathways without modifying cellular viabilities (90%). These encouraging results motivated these authors to propose the aforementioned niosome formulations as promising non-viral vehicles for plasmid DNA in vivo as EGFP expression can be evaluated in primary cortical embryos and blood cells [80].

The same research group studied other synthetic cationic lipids and helper lipids. Helper lipids have been anchored to liposome and niosome formulations with the aim of improving circulation in the bloodstream, stability and transfection potency [23,29,81]. There are a good number of helper lipids that have been commonly used in vitro and in vivo [e.g., DOPE (1,2-dioleyl-*sn*-glycero-3-phosphoethanolamine), Chol, DOPC (1,2-dioleyl-*sn*-glycero-3-phospho-choline) and PEG-C-DMA [3-*N*-[(ω-methoxypoly(ethylene glycol)2000carbamoyl]-1,2-dimyristyloxy-propylamine], among others]. An interesting study was carried out by Ojeda et al. who researched the impact of the amino lipid derivative **13** on the transfection process when combined with various helper lipids like squalene (SQ), Chol and squalane (SQL) [30]. The resultant cationic nioplexes were fully characterized and showed that SQL-based formulations displayed higher ζ-potential and particle size values than their niosome counterparts. However, cationic formulations containing SQ in their composition demonstrated superior transfection efficiencies and consequently, to promote cellular uptake through a micropinocytosis pathway which circumvents the degradation into the lysosomal compartment.

**Figure 5:**
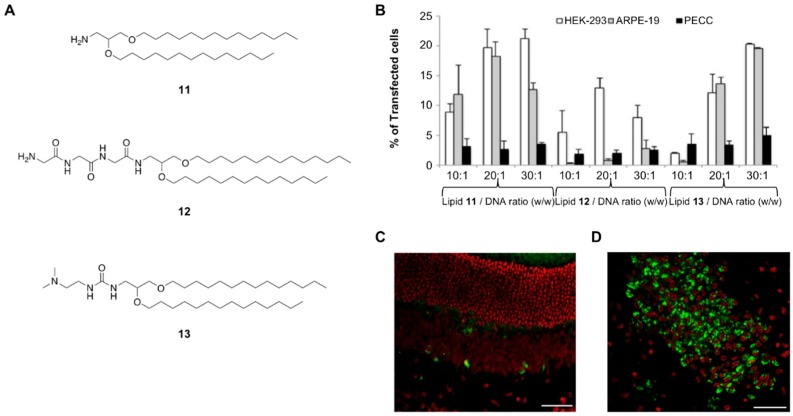
(**A**) Preparation of cationic niosome-based formulations containing lipids **11**, **12** and **13**, respectively and using SQ as a helper lipid; (**B**) in vitro gene transfection experiments after a 72-h incubation period at several mass ratios in three cell lines: HEK-293, ARPE-19 and PECC cells; (**C**) EGFP gene expression in retina after subretinal injection; (**D**) EGFP gene expression in the retina after intravitreal injection. Adapted with permission from ref. [82]. Copyright 2016, Elsevier B.V.

In light of these findings, Ojeda et al. carried out SAR studies by designing novel synthetic amino lipids (**11**–**13**) containing three different cationic polar heads such as an amino (**11**), triglycine peptide (**12**) and dimethylamino ethyl (**13**) pendent groups (Figure 5A) [82]. These three cationic lipids were formulated into the corresponding cationic niosomes in which SQ was part. After confirming that all of them were able to form nioplexes when mixed with a DNA plasmid (pCMS-EGFP), several physicochemical studies were carried out. It is worth mentioning that nioplex formation differed from the process used, thus the oil-in-water (O/W) emulsion technique led to lower polydispersity (PDI) values when compared to the film-hydration method. Interestingly, the prepared nioplexes displayed different a stability behaviour at different temperatures. Thus, while particle size and ζ-potential remained constant at 4 °C for 100 days, these parameters changed remarkably after incubating the nioplexes at 25 °C showing particle stabilities after less than one month. In vitro transfection experiments mediated by these novel nioplexes confirmed that the ionizable cationic lipid **11** and **13** promoted better efficiencies than formulations based on the tripeptide in ARPE-19 and PECC cells at the optimized 30:1 mass ratio (Figure 5B). These promising results were also demonstrated in vivo experiments by introducing both intravitreal (Figure 5C) and subretinal (Figure 5D) injections showing EGFP expression in photoreceptors and in the outer nuclear layer as well as in cerebral cortex administration in rat brains.

In addition to SQ and others helper lipids, alternative components have been introduced into niosome formulations with the aim of evaluating their transfection efficiencies. Protamine is a polycationic peptide that has been used in a good number of liposomal formulations in order to improve DNA condensation and enhance DNA delivery [36]. Puras et al. carried out this study by mixing protamine with a mixture containing Tween-80 and the corresponding ionizable amino lipid **11**. Optimal ratios for this ternary formulation were found at 1:1:5 (protamine:DNA:niosome) and it was used to deliver pEGFP plasmid in vitro using ARPE-19 cells also in vivo. The authors showed that the presence of protamine considerably improved the number of positive cells (26 %) when this transfection experiment was extended to 72 h. Although the value was lower than gene transfections mediated by lipofectamine (50% of positive cells), the authors reported cellular viabilities of up to 94% as well as good cell morphologies when the ternary system was used. This confirmed the potential use of this formulation in gene transfer into the retina. Finally, the authors studied the ability of this ternary system in order to mediate gene delivery in vivo. Interestingly, EGFP expression was observed in various cell retina layers depending on whether subretinal or intravitreal administrations were used.

In addition to adding helper lipids to niosome formulations, Puras et al. studied the efficiency of cationic niosomes based on cationic lipids (**11**) but removing the use of any helper lipid from the formulation [34]. Interestingly, niosomes exhibited the same ability to interact with DNA plasmid at various mass ratios. In this particular case, the authors found nanometric sizes (200 nm) and positive zeta potential values (7.3 and 13.2 mV at 2:1 and 6:1 mass ratio, respectively). Taking these values into account, the authors designed both in vitro and in vivo experiments using the corresponding nioplexes at an optimal ratio of 2:1. This ratio exhibited high efficiencies of transfection and cellular viabilities when compared to commercially available cationic lipids in two cell lines: HEK293 and ARPE-19. The ability of these nioplexes to impart cellular uptake was also demonstrated in vivo by carrying out subretinal and intravitreal injections and thus confirming the great potential of the cationic lipid **11** for retinal gene delivery therapies.

Additionally, the versatility of this formulation was also shown for bone regeneration applications by preparing the corresponding nioplexes from pUNO1-hBMP-7 plasmid and delivering it into mesenchymal stem cells (D1-MSCs) [83]. Preliminary results demonstrated the ability of nioplexes to impart cellular uptake in D1-MSCs (1460 pg/mL) at different mass ratios (4:1, 8:1 and 12:1, respectively) which produced a whole series of biological processes like cellular proliferation (164, 137 and 122% for 8:1, 12:1 and 16:1 mass ratio, respectively and alkaline phosphatase improvement facilitating osteoblast-like cell formation.

**Figure 6:**
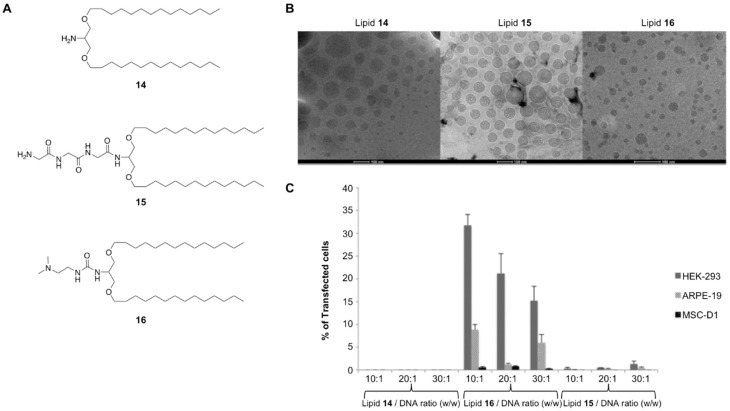
(**A**) Serinol-based cationic lipids that were formulated into cationic niosomes; (**B**) physical characterization by dynamic light scattering (DLS) showing spherical nanoscale patterns. Scale bar: 100 nm; (**C**) in vitro transfection efficiencies mediated using three serinol lipids. Adapted with permission from ref. [46]. Copyright 2015, The Royal Society of Chemistry.

Glycerol-based cationic lipids have proved to be efficient vehicles for transporting DNA plasmids, in particular lipids **11** and **13**. The impact of the amino lipids’ backbone on gene transfection was also studied in depth (Figure 6). Ojeda et al. proposed the use of serinol-based amino lipids (**14**--**16**) as novel vehicles to impart cellular uptake [46]. This study was aimed at synthesizing a small series of amino lipid derivatives containing the same cationic head pendent groups and hydrophobic residues as illustrated on Figure 5 but varying the backbone structure (Figure 6A). The oil-in-water emulsion technique gave rise to the corresponding cationic niosome formulations with nanometric size and lower PDI values when compared to the film-hydration approach (Figure 6B). Surprisingly, the authors found significant differences depending on the cationic lipid polar group after promoting cellular internalization. Thus, serinol-based amino lipids containing the ionizable dimethylamino ethyl residue (**16**) was able to transfect efficiently HEK-293 and ARPE-19 cells providing greater internalization results at 10:1 and 30:1 as an optimal mass ratio, (respectively) without affecting cellular proliferation (Figure 6C).

## 4. Use of Niosomes for Transfection of Oligonucleotides

### 4.1. Antisense Oligonucleotides

Antisense oligonucleotides (ASOs) are DNA oligonucleotides of around 18-mer length in which one of the non-bridging oxygens (PO) was replaced with phosphorothioate units (PS). The introduction of such modifications is critical in stabilizing oligonucleotides in the presence of nucleases over time [84]. There is growing interest in designing efficient ASOs against different types of cancer, diabetes and Duchenne muscular dystrophy among many others [57]. This great effort has contributed to launching several antisense-based therapeutics into the market like Fomivirsen, Mipomersen, Eteplirsen and Nusinersen as well as having many others in late clinical trials [3,85].

While using liposomes and solid lipid nanoparticles (SLNs) have been widely employed as non-viral carriers for ASOs [86,87,88,89], examples involving cationic niosomes are scarcely reported in literature. One of the first articles focused on promoting the delivery of ASOs with niosomes was described by Liang et al. in 2006 [90]. These authors prepared niosomes made up of commercially available cationic lipid (DC-Chol) and soybean phospholipids (SPLs) in the presence of sorbitan monoester series (Span-85, -80, -40 and -20) at several molar ratios using the film-hydration method. This protocol generated spherical particles in the nanometric size that ranged from 147 to 105 nm. After electrostatically complexing an antisense oligonucleotide and characterizing the corresponding particles using agarose electrophoresis, the authors carried out cellular uptake studies on COS-7 and HeLa cells. This demonstrated that some sorbitan fatty acids, in particular Span-40, have a pivotal role in promoting cellular internalization as well as showing some activity on gene expression. These results allowed the authors to modify the composition of these cationic niosomes by adding a modified polyethylene glycol (PEG2000-DSPE, 1,2-distearoyl-*sn*-glycero-3’-phospho- ethanolamine-*N*-[amino(polyethylene glycol)-2000 ammonium salt) instead of SPLs [35]. The presence of PEGylated niosomes favoured the interaction with protein sera and thus avoided particle aggregation after incubating the corresponding particles at sera concentrations of 0 and 50%, respectively. This result, though interesting, showed that the ability of these particles to promote cellular uptake was highly compromised since the particles were not able to facilitate endosomal escape as the concentration of DOPE was relatively low (up to 10% mol). This behaviour was confirmed using cetyltrimethylammonium ammonium bromide (CTAB) in the niosomal formulation, a cationic surfactant molecule that is able to facilitate endosomal escape [91].

**Figure 7:**
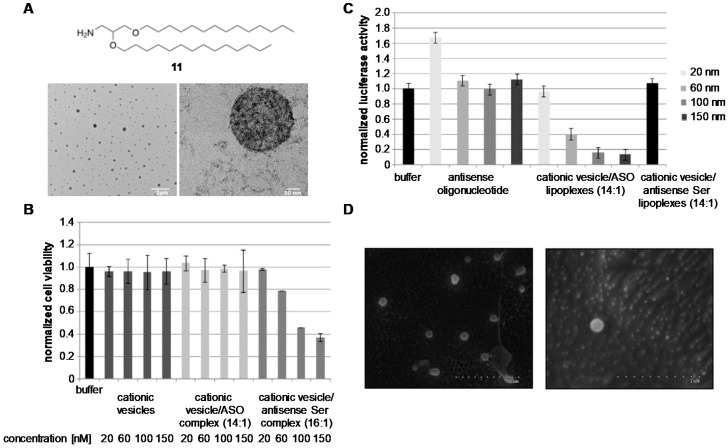
(**A**) Particle size characterization by DLS showing spherical nanosize pattern made up of the cationic lipid **11**; (**B**) Cellular viabilities of cationic niosomes and nioplexes at different concentrations and N/P ratios; (**C**) Dual-luciferase activity of cationic nioplexes at several concentrations (20, 60, 100 and 150 nM); (**D**) cryo-SEM observations showing cationic nioplexes encapsulated within tridimensional networks made up of κ-carrageenan. Adapted with permission from ref. [92,94]. Copyright 2014, Elsevier B.V. and Copyright 2017, The Royal Society of Chemistry.

In addition to displaying excellent efficiencies for the delivery of DNA plasmid, our research group decided to use synthetic amino lipid **11** to prepare cationic niosomes made up of non-ionic surfactant Tween-80 in order to prepare the corresponding nioplexes with a model antisense oligonucleotide (Figure 7) [92]. Interestingly, the expected nioplexes displayed spherical morphologies with nanometric sizes (324 and 332 nm at optimized N/P ratios of 14 and 16, respectively) (Figure 7A). Toxicity studies confirmed that N/P ratios greater than 16 had a strong negative effect on cellular proliferation (Figure 7B). To avoid this, gene transfection experiments were carried out in vitro at N/P ratios of 14 confirming the ability of cationic lipid **11** to silence luciferase efficiently [93] with comparable efficiencies to commercially available cationic lipids (approximately 85% of inhibition at 100 nM) (Figure 7C).

Further studies also confirmed that polysaccharide-based hydrogels made of κ-carrageenan proved to be appropriate tridimensional materials for holding the same cationic nioplexes described before without altering their physical properties (Figure 7D) [94]. The stability of such nioplexes within this tridimensional network was measured using cryo-SEM observation. The in vitro niosomal release was studied and confirmed that nioplexes were released? from the biodegradable material following Fickian diffusion controlled mechanisms as previously observed with other materials (i.e., phenylalanine-based supramolecular hydrogels) [95]. Interestingly, in vitro dose-response transfection experiments mediated by encapsulated nioplexes demonstrated significant silencing activities after 48-h of incubation (up to 51% at 300 nM) when compared to gene transfections mediated by the same hybrid material when incubated for only 24 h. These results opened up the possibility of using biodegradable polymers as non-toxic macroscopic materials that can be used in promoting the delivery of nucleic acids [96,97].

### 4.2. Aptamers

Aptamers are single stranded oligonucleotides that are able to fold and bind to a large variety of targets including proteins or small molecules [98]. These compounds are developed by selecting combinatorial nucleic acids libraries (SELEX). A large number of aptamers with a high affinity to proteins overexpressed in cancer cells have been found [98,99]. These are being used to decorate niosomes that have been used to entrap drugs for cancer treatment and provide excellent targeting capabilities for selective delivery to cancer cells.

One of the most efficient aptamers is the aptamer S2.2 (5′-GCA GTT GAT CCT TTG GAT ACC CTG G-3′) with a high affinity to the transmembrane glycoprotein MUC1 [100,101]. MUC1 protein is overexpressed in large number of malignant adenocarcinoma found in lung, ovarian, prostate, breast and ovarian cancers. This aptamer was selected by Seleci et al. [102] to functionalize niosomes loaded with Doxorubicin (DOX), a known inhibitor of DNA topoisomerases used in the treatment of malignant adenocarcinoma. The non-ionic vesicles named as PEGNIO were prepared by film hydratation followed by extrusion of niosomes constituted by Span 60, cholesterol, 1,2-distearoyl-*sn*-glycero-3-phosphoethanolamine-*N*-maleimide polyethyleneglycol 2000 (DSPE-PEG 2000 Maleimido) and doxorubicin. The aptamer carrying an amino group was crosslinked with Cys-TAT peptide (CYGRKKRRQRRR-NH2) and the resulting Cys-peptide-oligonucleotide conjugate was loaded into the niosomes using a thiol-maleimido reaction. The resulting DOX-PEGNIO-aptamer niosomes were more cytotoxic in HeLa cells because of the overexpression of MUC1 receptors demonstrating the successful targeting of niosomes by aptamers with affinity to overexpressed receptor proteins.

Another aptamer that was used to direct niosomes to cancer cells is aptamer AS1411. This nucleic acid aptamer has a strong affinity for nucleolin, a multifunctional protein overexpressed in most cancer cells [103]. This G-rich aptamer with the sequence 5′-GGT GGT GGT GGT TGT GGT GGT GGT GG-3′ has been used in Phase II clinical trials and it is commonly used for directing nanoparticles to cancer cells. Recently, Riccardi et al. has prepared niosomes loaded with the nucleolipid Ru(III) complex HoThyRu, a cytotoxic drug [104]. The niosomes made up ofTween-80, 2,3-bis(tetradecyloxy)propan-1-amine (DTPA) and HoThyRu were prepared using the film hydration approach. The addition of the AS1411 aptamer by electrostatic interaction with the cationic niosome induced a clear increase in the antiproliferative activity of niosomes loaded into HoThyRu in those cancer cells that are able to overexpress nucleolin [105].

### 4.3. siRNA and microRNA

RNA interference is a natural post-transcriptional gene regulation mechanism that was described for the first time in 1998 [106]. The effector molecules are double stranded RNA molecules with 21 bases (known as small-interfering RNAs, siRNAs) that bind to the RNA interference silencing complex (RISC) and the complexes are capable of selectively cleaving the complementary mRNA to one of the strands. Their high mRNA cleaving efficiency and the large turnover of the RISC-RNA complexes provide excellent properties for therapeutic use of siRNAs and a large number of them have been studied for the treatment of various diseases [107]. Recently, the first siRNA molecule, Partisiran, was approved for human use [5] but a lot of effort is still needed for the development of efficient delivery systems to improve the efficacy of the treatments. One of the most promising directions is the development of cationic niosomes for siRNA delivery.

One of the first studies on the use of non-ionic surfactants in RNA interference was the development of SPANosomes [108]. In this study, the surfactant Span-80 and the cationic lipid DOTAP were used to prepare cationic niosomes through the ethanol injection method along with adding 1–5% of d-α-tocopheryl PEG 1000 succinate to avoid RES uptake [108]. The resulting vesicles were unilamellar with a particle size smaller 50 nm and allowed high loading of siRNA forming complexes with good colloidal stability. The silencing activity of the SPANosomes/siRNA complexes was compared to the silencing activity of standard cationic liposomes/siRNA formulations resulting in higher silencing activity with results of 66–77% knock-down for niosome formulations. Through a careful analysis of the intracellular trafficking of the formulations, the authors were able to demonstrate that though cationic liposomes formulations, most of the liposomal vesicles were more abundant inside the cell and located in endosomes. In the case of formulations containing Span-80, they were able to escape endosomal compartments and were located in the cytoplasm [108,109].

Non-ionic surfactant vesicles (NISV) were also prepared using microfluidics for delivery of siRNA in cancer cell lines [110]. Obeid et al. studied the use of non-ionic surfactants monopalmotin glycerol (MPG) and polyoxyethylene sorbitol trioleate (Tween-85) mixed with the helper lipid cholesterol and DDAB as a cationic lipid. The authors found that small vesicles were obtained with excellent siRNA loading capacity, stability and low toxicity. But only formulations containing Tween-85 were able to transfect anti-green fluorescent protein (GFP) siRNA with 70% transfection efficiency similar to the efficiency obtained with cationic Hiperfect transfecting reagent [110]. Recently NISV were evaluated in a in vivo mice model with good results [111].

One of the most exciting directions in the study of the niosomes and siRNAs is the possibility of loading multiple drugs into niosomes in order to increase efficacy in cancer treatments. Sun et al. described the simultaneous delivery of DOX along with two siRNAs directed at inhibiting drug resistance mechanisms of cancer stem cells (CSCs) [112]. These authors selected siRNAs designed to inhibit ATP-binding cassette transporter (ABCG2) involved in multi-drug resistance and the anti-apoptotic protein BCL2 [112]. Niosomes were prepared using the ethanol injection method mixing the surfactant Span-80 and the cationic lipid DOTAP. Tocopherol-PEG 1000 succinate (TGPS) was added at 5% in order to avoid the removal of RES. DOX was dissolved in ethanol and loaded into the niosome during the ethanol injection process. The incorporation of the two siRNA molecules occurred through electrostatic interactions of the cationic niosomes with negatively charged RNAs. An enhanced cytotoxicity on both CSCs and parental cells was observed for the nioplexes carrying DOX and siRNAs. The authors were able to demonstrate that the activity mediated by DOX and siRNA complexes was connected to an increased apoptosis resulting from the down regulation of ABCG2 and BCL2 genes involved in the multi-drug resistance mechanisms [112].

Another study along those same lines described the synthesis of multilamellar niosomes carrying thymoquinone and a siRNA against the serine-threonine protein kinase Akt gene involved in tamoxifen-resistance [113]. Thymoquinone is a natural product obtained from black seed oil with significant antineoplastic activity. Overexpression of the Akt gene is involved in cell proliferation, migration and invasion. The multilamellar niosomes were synthesized by mixing Tween-80 and PEG. The cationic lipid was substituted with gold nanoparticles capped with octadecylamine (ODA). The presence of ODA afforded the positive charge needed for the complexation with siRNA. The resulting nioplexes provided stable to neutral conditions but were dissociated in an acidic medium present in tumours and endosomes. The efficacy of these nioplexes was also tested in tamoxifen-resistant and Akt—overexpressing MCF-7 breast cancer cells demonstrating that gold niosomes carrying the siRNA against Akt were more cytotoxic and displayed large apoptotic activity. These excellent results were confirmed in an in vivo study using MCF-7/TAM human breast carcinoma xenografts on mice. The gold niosomes carrying anti-Akt siRNA decreased the size of the tumour in the mice model thereby demonstrating the therapeutic potential of these niosomes [113].

A triple combination of drugs in niosomes has also been described by Hemati et al. [114]. In this study, DOX was encapsulated with the chemosensitizer quercetin. Quercitin is a phenolic compound with antioxidant and anti-proliferative properties. In addition to DOX and quercetin, a siRNA designed to inhibit the cell division cycle 20 (CDC20) protein was selected. This protein is involved in the cell-cycle checkpoint (G2/M) promoting the anaphase initiation. Overexpression of CDC20 was observed in cancer cells causing cell proliferation. The niosome formulation used in this study was prepared using thin-film hydration and sonication. Niosomes contained a mixture of the non-ionic surfactant Tween-60, Chol as a neutral helper lipid, the cationic lipids DOTAP and DPPC as well as 5% of DSPE-PEG2000. Quercitin was added to the lipid mixture before evaporating the solvents. However, DOX was added in the aqueous phase during the hydration step. Encapsulation efficiency was estimated to be around 90%. The siRNA was bound to the cationic PEGylated niosomes by electrostatic interactions. The optimal conditions for forming the nioplexes was found to be around a ratio of 40:1 (niosome/siRNA). Cytotoxicity assays in three cancer cell lines showed a higher toxicity of the niosome formulated drugs when compared to non-encapsulated drugs. In a non-cancer cell line the niosomal formulations were less toxic than the free drugs [114].

Recently, niosomes carrying nucleic acids therapeutic drugs and an activable near-infrared fluorescent dye were described [28]. This combination of treatment and tracking of stem cells into a single unit is known as theranostics. Yang et al. developed niosomes made up of surfactant Span-80 and the cationic lipid DOTAP and TPGS through the ethanol injection method in order to encapsulate indocyanine green (ICG). This is a tricarbocyanine dye approved by the FDA for clinical use with near-infrared emission properties [28]. The niosomes carrying the indocyanine dye (iSPN) were functionalized with nucleic acids such as siRNA and anti-microRNA through electrostatic interactions. MicroRNA are small endogenous RNAs that are produced to control gene expression [115]. The dysregulation of microRNA may cause diseases. When critical miRNAs are overexpressed, one may use complementary oligonucleotides to inhibit these particular miRNAs. These nucleic acids are known as anti-miRNA or antagomirs [116]. Yang et al. tested the iSPN to transfect siRNA and anti-miRNA into mesenchymal stem cells (MSCs) and imaging these cells in vivo. The authors demonstrated that iSPN was able to transfect anti –GFP siRNA and induced 85–88% silencing efficiency in optimal loading conditions with no apparent cytoxicity. When iSPN were loaded into anti-miRNA-138, the resulting nioplexes were able to accelerate osteogenic differentiation of human MSCs as expected for the inhibition of overexpressed miRNA-138. Moreover, iSPN was activated upon cellular internalization becoming very fluorescent allowing stem cells to be labelled and facilitating the observation of their distribution in live animals [28]. To summarize, all the examples shown in this manuscript that cover the use of niosome-based formulations with the aim at transporting genetic material (plasmid DNA, ASOs, siRNAs and aptamers) and consequently displaying their biocompatible and minimal toxicities are listed in Table 2.

## 5. Conclusions and Future Perspectives

Colloidal vesicles have become a useful approach for facilitating cellular transport not only for small molecule drugs but also for macromolecule therapeutics like nucleic acids, antibodies and proteins. Since the discovery of antisense technology and RNA interference mechanisms and with the recent FDA-approval of Patisiran as the first RNAi therapeutic drug in addition to other approved oligonucleotide therapies [3], the use of nucleic acids has opened up promising horizons in the search for novel drugs. While covalent strategies [7] have enabled is to modify both the termini as well as intermediate positions of ASO, siRNA or miRNA molecules with cholesterol [117], fatty acids [118], *N*-acetylgalactosamine derivatives [119] and polymers [120] among others, the development of appropriate formulations is still required for transporting a large number of cargoes to specific cell tissues and organs. In fact, optimized formulations have allowed chemotherapeutic molecules like DOX to increase their potency and activity against certain solid tumours [121]. This knowledge has contributed to relaunching the potential of nucleic acids as therapeutics by placing powerful formulations on the market [5] and in late clinical studies [122].

Despite the success and extensive use of liposomes in therapy [123], a large number of drug delivery systems based on synthetic cationic lipids have presented themselves as alternatives and have received a lot of attention. Niosomes are a type of vesicles made up of hydrated non-ionic surfactant molecules. They have emerged as a potential drug delivery system since they have shown similar structural features to liposomes in addition to their apparent ability to entrap different types of cargoes. This comparable behaviour includes the ease of manufacturing and combining their long-term stabilities which have allowed niosomes to be used in a plethora of biomedical therapies.

While transporting DNA plasmids into target cells and evaluating their efficiency in vivo have been widely described by synthesizing novel cationic lipids and preparing non-ionic surfactant-based formulations, recent advances in genome engineering with RNA-guide CRISPR-Cas9 system [124,125] might open up new horizons for using such non-viral vectors. In fact, some liposome-based and polymeric materials have been proposed favouring their delivery and therefore enabling genome editing with promising results [126].

Another important point to take into consideration concerns developing safe and effective formulation strategies for other nucleic acid therapeutics (e.g., ASOs, siRNAs, miRNAs and mRNAs). Notwithstanding, a few interesting approaches have been developed using niosomes, alternative colloidal systems like liposomes though solid lipid nanoparticles continue to be the leading choice for administering the aforementioned cargoes in vivo. As with the CRISPR-Cas9 system, further and detailed research involving non-ionic surfactant formulations and nucleic acids are necessary and may involve novel therapeutic approaches as well as provide considerable opportunities to bring more oligonucleotide-based therapeutics to the market.

## Figures and Tables

**Table 1:** Particle size, optimal weight ratio and transfection efficiency for cationic nioplexes made up of DNA plasmid and a mixture of spermine derivatives, Chol and Span-20.

Spermine-Based Cationic Lipid	Particle Size (nm)	Optimal Weight Ratio	Transfection Efficiency (cells/cm^2^)
1	213	10	7556 ± 92
2	315	10	6897 ± 292
3	487	5	5453 ± 36
4	876	20	2082 ± 63
5	462	30	5959 ± 197
6	385	10	7993 ± 94

**Table 2:** Niosome-based non-viral vehicles designed to delivery genetic material inside cells.

Cationic Lipid	Niosome Preparation	Cargo	Therapy	Testing Conditions	References
Polyamine derivatives	Thin-film	pEGFP-C2	-	In vitro	[68,71]
Polyamine derivative	Thin-film	pOVA	Skin vaccination	In vivo	[72]
DODAB	Thin-film	pMEL34 and pLuc	Topical delivery	In vitro	[74,75]
DOTMA	Reverse-phase evaporation	pCMS-EGFP	Ocular delivery	In vitro and In vivo	[78]
13	Oil-in-water emulsion	pCMS-EGFP	-	In vitro	[30]
Glycerol-based amino lipid derivatives	Oil-in-water emulsion	pCMS-EGFP	Ocular delivery	In vitro and In vivo	[82]
DTPA	Emulsification-evaporation	pCMS-EGFP	Ocular delivery	In vitro and In vivo	[34]
DTPA	Reverse-phase evaporation	pUNO1-hBMP-7	Bone regeneration	In vitro	[83]
Serinol-based amino lipid derivatives	Oil-in-water emulsion	pCMS-EGFP	-	In vitro	[46]
DC-Chol	Reverse-phase evaporation and thin-film	ASO	-	In vitro	[90,91]
DTPA	Thin-film	ASO	-	In vitro	[92]
PEGNIO	Thin-film	MUC1 Aptamer	Chemotherapy	In vitro	[102]
DTPA	Thin-film	AS1411	Chemotherapy	In vitro	[105]
DOTAP	Ethanol injection	siLuc	-	In vitro	[108]
DDAB	Microfluidic	siRNA GFP	Chemotherapy	In vitro and In vivo	[110,111]
DOTAP	Ethanol injection	2 siRNAs	Chemotherapy	In vivo	[112]
Gold niosomes (Nio-Au)	Ethanol evaporation	siRNA	Chemotherapy	In vivo	[113]
DOTAP	Thin-film	siRNA	Chemotherapy	In vitro	[114]
DOTAP	Ethanol injection	siRNA and miRNA	Chemotherapy	In vitro and In vivo	[28]

## References

[1] Collins M., Thrasher A. (2015). Gene therapy: Progress and predictions. Proc. R. Soc. B Biol. Sci..

[2] Bennett C.F., Baker B.F., Pham N., Swayze E., Geary R.S. (2017). Pharmacology of Antisense Drugs. Annu. Rev. Pharmacol. Toxicol..

[3] Stein C.A., Castanotto D. (2017). FDA-Approved Oligonucleotide Therapies in 2017. Mol. Ther..

[4] Deleavey G.F., Damha M.J. (2012). Designing chemically modified oligonucleotides for targeted gene silencing. Chem. Biol..

[5] Adams D., Gonzalez-Duarte A., O’Riordan W.D., Yang C.-C., Ueda M., Kristen A.V., Tournev I., Schmidt H.H., Coelho T., Berk J.L. (2018). Patisiran, an RNAi Therapeutic, for Hereditary Transthyretin Amyloidosis. N. Engl. J. Med..

[6] Kim M., Kim D.M., Kim K.S., Jung W., Kim D.E. (2018). Applications of cancer cell-specific aptamers in targeted delivery of anticancer therapeutic agents. Molecules.

[7] Grijalvo S., Alagia A., Jorge F.A., Eritja R. (2018). Covalent Strategies for Targeting Messenger and Non-Coding RNAs: An Updated Review on siRNA, miRNA and antimiR Conjugates. Genes.

[8] Oh Y.K., Park T.G. (2009). siRNA delivery systems for cancer treatment. Adv. Drug Deliv. Rev..

[9] Giacca M., Zacchigna S. (2012). Virus-mediated gene delivery for human gene therapy. J. Control. Release.

[10] Raouane M., Desmaële D., Urbinati G., Massaad-Massade L., Couvreur P. (2012). Lipid Conjugated Oligonucleotides: A Useful Strategy for Delivery. Bioconjug. Chem..

[11] Trabulo S., Cardoso A.L., Cardoso A.M.S., Morais C.M., Jurado A.S., de Lima M.C. (2013). Cell-penetrating Peptides as Nucleic Acid Delivery Systems: From Biophysics to Biological Applications. Curr. Pharm. Des..

[12] Wagner E. (2012). Polymers for siRNA delivery: Inspired by viruses to be targeted, dynamic and precise. Acc. Chem. Res..

[13] Ghosh P., Han G., De M., Kim C.K., Rotello V.M. (2008). Gold nanoparticles in delivery applications. Adv. Drug Deliv. Rev..

[14] Movahedi F., Hu R.G., Becker D.L., Xu C. (2015). Stimuli-responsive liposomes for the delivery of nucleic acid therapeutics. Nanomed. Nanotechnol. Biol. Med..

[15] Templeton N.S. (2002). Liposomal delivery of nucleic acids in vivo. DNA Cell Biol..

[16] Akinc A., Goldberg M., Qin J., Dorkin J.R., Gamba-Vitalo C., Maier M., Jayaprakash K.N., Jayaraman M., Rajeev K.G., Manoharan M. (2009). Development of lipidoid-sirna formulations for systemic delivery to the liver. Mol. Ther..

[17] Leung A.K.K., Tam Y.Y.C., Cullis P.R. (2014). Lipid Nanoparticles for Short Interfering RNA Delivery.

[18] Semple S.C., Akinc A., Chen J., Sandhu A.P., Mui B.L., Cho C.K., Sah D.W.Y., Stebbing D., Crosley E.J., Yaworski E. (2010). Rational design of cationic lipids for siRNA delivery. Nat. Biotechnol..

[19] Durymanov M., Reineke J. (2018). Non-viral delivery of nucleic acids: Insight into mechanisms of overcoming intracellular barriers. Front. Pharmacol..

[20] Kim S.I., Shin D., Choi T.H., Lee J.C., Cheon G.J., Kim K.Y., Park M., Kim M. (2007). Systemic and specific delivery of small interfering RNAs to the liver mediated by apolipoprotein A-I. Mol. Ther..

[21] Paecharoenchai O., Teng L., Yung B.C., Opanasopit P., Lee R.J. (2013). Non-ionic surfactant vesicles for delivery of RNAi therapeutics. Nanomedicine.

[22] Mahale N.B., Thakkar P.D., Mali R.G., Walunj D.R., Chaudhari S.R. (2012). Niosomes: Novel sustained release nonionic stable vesicular systems—An overview. Adv. Colloid Interface Sci..

[23] Ojeda E., Agirre M., Villate-Beitia I., Mashal M., Puras G., Zarate J., Pedraz J.L., Candiani G. (2016). Elaboration and Physicochemical Characterization of Niosome-Based Nioplexes for Gene Delivery Purposes BT—Non-Viral Gene Delivery Vectors: Methods and Protocols.

[24] Kazi K.M., Mandal A.S., Biswas N., Guha A., Chatterjee S., Behera M., Kuotsu K. (2010). Niosome: A future of targeted drug delivery systems. J. Adv. Pharm. Technol. Res..

[25] Thakkar M., Brijesh S. (2016). Opportunities and Challenges for Niosomes as Drug Delivery Systems. Curr. Drug Deliv..

[26] Kaur G., Mehta S.K. (2017). Developments of Polysorbate (Tween) based microemulsions: Preclinical drug delivery, toxicity and antimicrobial applications. Int. J. Pharm..

[27] Moghassemi S., Hadjizadeh A. (2014). Nano-niosomes as nanoscale drug delivery systems: An illustrated review. J. Control. Release.

[28] Yang C., Gao S., Song P., Dagnæs-Hansen F., Jakobsen M., Kjems J. (2018). Theranostic Niosomes for Efficient siRNA/MicroRNA Delivery and Activatable Near-Infrared Fluorescent Tracking of Stem Cells. ACS Appl. Mater. Interfaces.

[29] Hui S.W., Langner M., Zhao Y.L., Ross P., Hurley E., Chan K. (1996). The role of helper lipids in cationic liposome-mediated gene transfer. Biophys. J..

[30] Ojeda E., Puras G., Agirre M., Zarate J., Grijalvo S., Eritja R., Digiacomo L., Caracciolo G., Pedraz J.L. (2016). The role of helper lipids in the intracellular disposition and transfection efficiency of niosome formulations for gene delivery to retinal pigment epithelial cells. Int. J. Pharm..

[31] Gao X., Huang L. (1991). A novel cationic liposome reagent for efficient transfection of mammalian cells. Biochem. Biophys. Res. Commun..

[32] Regelin A.E., Fankhaenel S., Gürtesch L., Prinz C., von Kiedrowski G., Massing U. (2000). Biophysical and lipofection studies of DOTAP analogs. Biochim. Biophys. Acta Biomembr..

[33] Dass C.R. (2004). Lipoplex-mediated delivery of nucleic acids: Factors affecting in vivo transfection. J. Mol. Med..

[34] Ochoa G.P., Sesma J.Z., Díez M.A., Díaz-Tahoces A., Avilés-Trigeros M., Grijalvo S., Eritja R., Fernández E., Pedraz J.L. (2014). A novel formulation based on 2,3-di(tetradecyloxy)propan-1-amine cationic lipid combined with polysorbate 80 for efficient gene delivery to the retina. Pharm. Res..

[35] Huang Y., Chen J., Chen X., Gao J., Liang W. (2008). PEGylated synthetic surfactant vesicles (Niosomes): Novel carriers for oligonucleotides. J. Mater. Sci. Mater. Med..

[36] Sorgi F.L., Bhattacharya S., Huang L. (1997). Protamine sulphate enhances lipid-mediated gene transfer. Gene Ther..

[37] Attia N., Soto C., Martínez G., Eritja R., Puras G., Luis J. (2018). Gene transfer to rat cerebral cortex mediated by polysorbate 80 and poloxamer 188 nonionic surfactant vesicles. Drug Des. Dev. Ther..

[38] Baillie A.J., Florence A.T., Hume L.R., Muirhead G.T., Rogerson A. (1985). The preparation and properties of niosomes—Non-ionic surfactant vesicles. J. Pharm. Pharmacol..

[39] Manosroi A., Wongtrakul P., Manosroi J., Sakai H., Sugawara F., Yuasa M., Abe M. (2003). Characterization of vesicles prepared with various non-ionic surfactants mixed with cholesterol. Colloids Surf. B Biointerfaces.

[40] Garcia-Salinas S., Himawan E., Mendoza G., Arruebo M., Sebastian V. (2018). Rapid on-Chip Assembly of Niosomes: Batch versus Continuous Flow Reactors. ACS Appl. Mater. Interfaces.

[41] Lo C.T., Jahn A., Locascio L.E., Vreeland W.N. (2010). Controlled Self-Assembly of Monodisperse Niosomes by Microfluidic Hydrodynamic Focusing. Langmuir.

[42] Alatorre-Meda M., Taboada P., Hartl F., Wagner T., Freis M., Rodríguez J.R. (2011). The influence of chitosan valence on the complexation and transfection of DNA: The weaker the DNA–chitosan binding the higher the transfection efficiency. Colloids Surf. B Biointerfaces.

[43] Pozharski E., MacDonald R.C. (2002). Thermodynamics of cationic lipid-DNA complex formation as studied by isothermal titration calorimetry. Biophys. J..

[44] Zhang J., Fan H., Levorse D.A., Crocker L.S. (2011). Ionization behaviour of amino lipids for siRNA delivery: Determination of ionization constants, SAR and the impact of lipid p K a on cationic lipid-biomembrane interactions. Langmuir.

[45] Jayaraman M., Ansell S.M., Mui B.L., Tam Y.K., Chen J., Du X., Butler D., Eltepu L., Matsuda S., Narayanannair J.K. (2012). Maximizing the potency of siRNA lipid nanoparticles for hepatic gene silencing in vivo. Angew. Chem. Int. Ed..

[46] Ojeda E., Puras G., Agirre M., Zárate J., Grijalvo S., Pons R., Eritja R., Martinez-Navarrete G., Soto-Sanchez C., Fernández E. (2015). Niosomes based on synthetic cationic lipids for gene delivery: The influence of polar head-groups on the transfection efficiency in HEK-293, ARPE-19 and MSC-D1 cells. Org. Biomol. Chem..

[47] Tristram-Nagle S., Nagle J.F. (2004). Lipid bilayers: Thermodynamics, structure, fluctuations and interactions. Chem. Phys. Lipids.

[48] Pack D.W., Hoffman A.S., Pun S., Stayton P.S. (2005). Design and development of polymers for gene delivery. Nat. Rev. Drug Discov..

[49] El-Serag H.B., Rudolph K.L. (2007). Hepatocellular Carcinoma: Epidemiology and Molecular Carcinogenesis. Gastroenterology.

[50] Caddeo C., Nacher A., Vassallo A., Armentano M.F., Pons R., Fernàndez-Busquets X., Carbone C., Valenti D., Fadda A.M., Manconi M. (2016). Effect of quercetin and resveratrol co-incorporated in liposomes against inflammatory/oxidative response associated with skin cancer. Int. J. Pharm..

[51] Pabst G., Kučerka N., Nieh M.-P., Rheinstädter M.C., Katsaras J. (2010). Applications of neutron and X-ray scattering to the study of biologically relevant model membranes. Chem. Phys. Lipids.

[52] Kucerka N., Nieh M.P., Katsaras J. (2009). Asymmetric distribution of cholesterol in unilamellar vesicles of monounsaturated phospholipids. Langmuir.

[53] Handjani-Vila R.M., Ribier A., Rondot B., Vanlerberghie G. (1979). Dispersions of lamellar phases of non-ionic lipids in cosmetic products. Int. J. Cosmet. Sci..

[54] Khan R., Irchhaiya R. (2016). Niosomes: A potential tool for novel drug delivery. J. Pharm. Investig..

[55] Khan R., Irchhaiya R. (2017). An overview on niosomes as efficient drug carriers. Int. J. Pharm. Biosci..

[56] Rajera R., Nagpal K., Singh S.K., Mishra D.N. (2011). Niosomes: A Controlled and Novel Drug Delivery System. Biol. Pharm. Bull..

[57] Chan J.H.P., Lim S., Wong W.S.F. (2006). Antisense oligonucleotides: From design to therapeutic application. Clin. Exp. Pharmacol. Physiol..

[58] Burnett J., Rossi J. (2012). RNA-Based Therapeutics: Current Progress and Future Prospects. Chem. Biol..

[59] Hajj K.A., Whitehead K.A. (2017). Tools for translation: Non-viral materials for therapeutic mRNA delivery. Nat. Rev. Mater..

[60] Baxter R., Hastings N., Law A., Glass E.J. (2008). Plasmids for Therapy and Vaccination; Animal Genetics.

[61] Li S.D., Huang L. (2006). Gene therapy progress and prospects: Non-viral gene therapy by systemic delivery. Gene Ther..

[62] Zhi D., Zhang S., Cui S., Zhao Y., Wang Y., Zhao D. (2013). The Headgroup Evolution of Cationic Lipids for Gene Delivery. Bioconjug. Chem..

[63] Teixeira H.F., Bruxel F., Fraga M., Schuh R.S., Zorzi G.K., Matte U., Fattal E. (2017). Cationic nanoemulsions as nucleic acids delivery systems. Int. J. Pharm..

[64] Felgner P.L., Gadek T.R., Holm M., Roman R., Chan H.W., Wenz M., Northrop J.P., Ringold G.M., Danielsen M. (1987). Lipofection: A highly efficient, lipid-mediated DNA-transfection procedure. Proc. Natl. Acad. Sci. USA.

[65] Miller A.D. (1998). Cationic Liposomes for Gene Therapy. Angew. Chem. Int. Ed..

[66] Lewis J.G., Lin K.-Y., Kothavale A., Flanagan W.M., Matteucci M.D., DePrince R.B., Mook R.A., Hendren R.W., Wagner R.W. (1996). A serum-resistant cytofectin for cellular delivery of antisense oligodeoxynucleotides and plasmid DNA. Proc. Natl. Acad. Sci. USA.

[67] Massing U., Kley J.T., Gürtesch L., Fankhaenel S. (2000). A simple approach to DOTAP and its analogs bearing different fatty acids. Chem. Phys. Lipids.

[68] Ronsin G., Perrin C., Guédat P., Kremer A., Camilleri P., Kirby A.J. (2001). Novel spermine-based cationic gemini surfactants for gene delivery. Chem. Commun..

[69] Paecharoenchai O., Niyomtham N., Leksantikul L., Ngawhirunpat T., Rojanarata T., Yingyongnarongkul B., Opanasopit P. (2014). Non-ionic Surfactant Vesicles Composed of Novel Spermine-Derivative Cationic Lipids as an Effective Gene Carrier In Vitro. AAPS PharmSciTech.

[70] Paecharoenchai O., Niyomtham N., Ngawhirunpat T., Rojanarata T., Yingyongnarongkul B.E., Opanasopit P. (2012). Cationic niosomes composed of spermine-based cationic lipids mediate high gene transfection efficiency. J. Drug Target..

[71] Opanasopit P., Leksantikul L., Niyomtham N., Ngawhirunpat T., Yingyongnarongkul B., Opanasopit P., Leksantikul L., Niyomtham N., Rojanarata T., Ngawhirunpat T. (2015). Cationic niosomes an effective gene carrier composed of novel spermine-derivative cationic lipids: Effect of central core structures Cationic niosomes an effective gene carrier composed of novel spermine-derivative cationic lipids: Effect of central cor. Pharm. Dev. Technol..

[72] Pamornpathomkul B., Niyomtham N., Yingyongnarongkul B.-E., Prasitpuriprecha C., Rojanarata T., Ngawhirunpat T., Opanasopit P. (2018). Cationic Niosomes for Enhanced Skin Immunization of Plasmid DNA-Encoding Ovalbumin via Hollow Microneedles. AAPS PharmSciTech.

[73] Rose J.K., Buonocore L., Whitt M.A. (1991). A new cationic liposome reagent mediating nearly quantitative transfection of animal cells. Biotechniques.

[74] Manosroi J., Khositsuntiwong N., Manosroi W., Götz F., Werner R.G., Manosroi A. (2010). Enhancement of Transdermal Absorption, Gene Expression and Stability of Tyrosinase Plasmid (pMEL34)-Loaded Elastic Cationic Niosomes: Potential Application in Vitiligo Treatment. J. Pharm. Sci..

[75] Manosroi A., Thathang K., Werner R.G., Schubert R., Manosroi J. (2008). Stability of luciferase plasmid entrapped in cationic bilayer vesicles. Int. J. Pharm..

[76] Conley S.M., Naash M.I. (2010). Nanoparticles for retinal gene therapy. Prog. Retin. Eye Res..

[77] Yin H., Kanasty R.L., Eltoukhy A.A., Vegas A.J., Dorkin J.R., Anderson D.G. (2014). Non-viral vectors for gene-based therapy. Nat. Rev. Genet..

[78] Mashal M., Attia N., Puras G., Martínez-Navarrete G., Fernández E., Pedraz J.L. (2017). Retinal gene delivery enhancement by lycopene incorporation into cationic niosomes based on DOTMA and polysorbate 60. J. Control. Release.

[79] Di Mascio P., Kaiser S., Sies H. (1989). Lycopene as the most efficient biological carotenoid singlet oxygen quencher. Arch. Biochem. Biophys..

[80] Mashal M., Attia N., Soto-Sánchez C., Martínez-Navarrete G., Fernández E., Puras G., Pedraz J.L. (2018). Non-viral vectors based on cationic niosomes as efficient gene delivery vehicles to central nervous system cells into the brain. Int. J. Pharm..

[81] Cheng X., Lee R.J. (2016). The role of helper lipids in lipid nanoparticles (LNPs) designed for oligonucleotide delivery. Adv. Drug Deliv. Rev..

[82] Ojeda E., Puras G., Agirre M., Zarate J., Grijalvo S., Eritja R., Fern E. (2016). The influence of the polar head-group of synthetic cationic lipids on the transfection ef fi ciency mediated by niosomes in rat retina and brain. Biomaterials.

[83] Attia N., Mashal M., Grijalvo S., Eritja R., Zárate J., Puras G., Pedraz J.L. (2018). Stem cell-based gene delivery mediated by cationic niosomes for bone regeneration. Nanomed. Nanotechnol. Biol. Med..

[84] Dias N., Stein C.A. (2002). Antisense oligonucleotides: Basic concepts and mechanisms. Mol. Cancer Ther..

[85] Kaczmarek J.C., Kowalski P.S., Anderson D.G. (2017). Advances in the delivery of RNA therapeutics: From concept to clinical reality. Genome Med..

[86] Jin S.E., Kim C.K. (2012). Long-term stable cationic solid lipid nanoparticles for the enhanced intracellular delivery of SMAD3 antisense oligonucleotides in activated murine macrophages. J. Pharm. Pharm. Sci..

[87] Li H., Quan J., Zhang M., Yung B.C., Cheng X., Liu Y., Lee Y.B., Ahn C.H., Kim D.J., Lee R.J. (2016). Lipid-albumin nanoparticles (LAN) for therapeutic delivery of antisense oligonucleotide against HIF-1α. Mol. Pharm..

[88] Siddiqui A., Gupta V., Liu Y.Y., Nazzal S. (2012). Doxorubicin and MBO-asGCS oligonucleotide loaded lipid nanoparticles overcome multidrug resistance in adriamycin resistant ovarian cancer cells (NCI/ADR-RES). Int. J. Pharm..

[89] Fattal E., Couvreur P., Dubernet C. (2004). “Smart” delivery of antisense oligonucleotides by anionic pH-sensitive liposomes. Adv. Drug Deliv. Rev..

[90] Huang Y.Z., Gao J.Q., Chen J.L., Liang W.Q. (2006). Cationic liposomes modified with non-ionic surfactants as effective non-viral carrier for gene transfer. Colloids Surf. B Biointerfaces.

[91] Timko B.P., Dvir T., Kohane D.S. (2010). Remotely triggerable drug delivery systems. Adv. Mater..

[92] Grijalvo S., Alagia A., Puras G., Zárate J., Pedraz J.L., Eritja R. (2014). Cationic vesicles based on non-ionic surfactant and synthetic aminolipids mediate delivery of antisense oligonucleotides into mammalian cells. Colloids Surf. B Biointerfaces.

[93] Zhang H.-Y. (2003). mRNA accessible site tagging (MAST): A novel high throughput method for selecting effective antisense oligonucleotides. Nucleic Acids Res..

[94] Grijalvo S., Alagia A., Puras G., Zárate J., Mayr J., Pedraz J.L., Eritja R., Díaz D.D. (2017). Cationic nioplexes-in-polysaccharide-based hydrogels as versatile biodegradable hybrid materials to deliver nucleic acids. J. Mater. Chem. B.

[95] Grijalvo S., Puras G., Zárate J., Pons R., Pedraz J.L., Eritja R., Díaz D.D. (2016). Nioplexes encapsulated in supramolecular hybrid biohydrogels as versatile delivery platforms for nucleic acids. RSC Adv..

[96] Krebs M.D., Jeon O., Alsberg E. (2009). Localized and sustained delivery of silencing RNA from macroscopic biopolymer hydrogels. J. Am. Chem. Soc..

[97] Grijalvo S., Mayr J., Eritja R., Díaz D.D. (2016). Biodegradable liposome-encapsulated hydrogels for biomedical applications: A marriage of convenience. Biomater. Sci..

[98] Zhou J., Rossi J. (2016). Aptamers as targeted therapeutics: Current potential and challenges. Nat. Rev. Drug Discov..

[99] Cerchia L., de Franciscis V. (2010). Targeting cancer cells with nucleic acid aptamers. Trends Biotechnol..

[100] Ferreira C.S.M., Matthews C.S., Missailidis S. (2006). DNA Aptamers That Bind to MUC1 Tumour Marker: Design and Characterization of MUC1-Binding Single-Stranded DNA Aptamers. Tumor Biol..

[101] Hu Y., Duan J., Zhan Q., Wang F., Lu X., Yang X.-D. (2012). Novel MUC1 Aptamer Selectively Delivers Cytotoxic Agent to Cancer Cells In Vitro. PLoS ONE.

[102] Seleci D.A., Seleci M., Jochums A., Walter J.G., Stahl F., Scheper T. (2016). Aptamer mediated niosomal drug delivery. RSC Adv..

[103] Bates P.J., Reyes-Reyes E.M., Malik M.T., Murphy E.M., O’Toole M.G., Trent J.O. (2017). G-quadruplex oligonucleotide AS1411 as a cancer-targeting agent: Uses and mechanisms. Biochim. Biophys. Acta Gen. Subj..

[104] Mangiapia G., D’Errico G., Simeone L., Irace C., Radulescu A., Di Pascale A., Colonna A., Montesarchio D., Paduano L. (2012). Ruthenium-based complex nanocarriers for cancer therapy. Biomaterials.

[105] Riccardi C., Fàbrega C., Grijalvo S., Vitiello G., D’Errico G., Eritja R., Montesarchio D. (2018). AS1411-decorated niosomes as effective nanocarriers for Ru(iii)-based drugs in anticancer strategies. J. Mater. Chem. B.

[106] Fire A., Xu S., Montgomery M.K., Kostas S.A., Driver S.E., Mello C.C. (1998). Potent and specific genetic interference by double-stranded RNA in Caenorhabditis elegans. Nature.

[107] Wittrup A., Lieberman J. (2015). Knocking down disease: A progress report on siRNA therapeutics. Nat. Rev. Genet..

[108] Zhou C., Mao Y., Sugimoto Y., Zhang Y., Kanthamneni N., Yu B., Brueggemeier R.W., Lee L.J., Lee R.J. (2012). SPANosomes as delivery vehicles for small interfering RNA (siRNA). Mol. Pharm..

[109] Zhou C., Zhang Y., Yu B., Phelps M.A., Lee L.J., Lee R.J. (2013). Comparative cellular pharmacokinetics and pharmacodynamics of siRNA delivery by SPANosomes and by cationic liposomes. Nanomed. Nanotechnol. Biol. Med..

[110] Obeid M.A., Elburi A., Young L.C., Mullen A.B., Tate R.J., Ferro V.A. (2017). Formulation of Non-ionic Surfactant Vesicles (NISV) Prepared by Microfluidics for Therapeutic Delivery of siRNA into Cancer Cells. Mol. Pharm..

[111] Obeid M.A., Dufès C., Somani S., Mullen A.B., Tate R.J., Ferro V.A. (2018). Proof of concept studies for siRNA delivery by non-ionic surfactant vesicles: In vitro and in vivo evaluation of protein knockdown. J. Liposome Res..

[112] Sun M., Yang C., Zheng J., Wang M., Chen M., Le D.Q.S., Kjems J., Bünger C.E. (2015). Enhanced efficacy of chemotherapy for breast cancer stem cells by simultaneous suppression of multidrug resistance and antiapoptotic cellular defense. Acta Biomater..

[113] Rajput S., Puvvada N., Kumar B.N.P., Sarkar S., Konar S., Bharti R., Dey G., Mazumdar A., Pathak A., Fisher P.B. (2015). Overcoming Akt Induced Therapeutic Resistance in Breast Cancer through siRNA and Thymoquinone Encapsulated Multilamellar Gold Niosomes. Mol. Pharm..

[114] Hemati M., Haghiralsadat F., Yazdian F., Jafari F., Moradi A., Malekpour-Dehkordi Z. (2018). Development and characterization of a novel cationic PEGylated niosome-encapsulated forms of doxorubicin, quercetin and siRNA for the treatment of cancer by using combination therapy. Artif. Cells Nanomed. Biotechnol..

[115] Cai Y., Yu X., Hu S., Yu J. (2009). A Brief Review on the Mechanisms of miRNA Regulation. Genom. Proteom. Bioinform..

[116] Gambari R., Brognara E., Spandidos D.A., Fabbri E. (2016). Targeting oncomiRNAs and mimicking tumour suppressor miRNAs: Ew trends in the development of miRNA therapeutic strategies in oncology. Int. J. Oncol..

[117] Wolfrum C., Shi S., Jayaprakash K.N., Jayaraman M., Wang G., Pandey R.K., Rajeev K.G., Nakayama T., Charrise K., Ndungo E.M. (2007). Mechanisms and optimization of in vivo delivery of lipophilic siRNAs. Nat. Biotechnol..

[118] Lorenz C., Hadwiger P., John M., Vornlocher H.P., Unverzagt C. (2004). Steroid and lipid conjugates of siRNAs to enhance cellular uptake and gene silencing in liver cells. Bioorgan. Med. Chem. Lett..

[119] Nair J.K., Willoughby J.L.S., Chan A., Charisse K., Alam M.R., Wang Q., Hoekstra M., Kandasamy P., Kel’in A.V., Milstein S. (2014). Multivalent N-Acetylgalactosamine-Conjugated siRNA Localizes in Hepatocytes and Elicits Robust RNAi-Mediated Gene Silencing. J. Am. Chem. Soc..

[120] Parmar R.G., Poslusney M., Busuek M., Williams J.M., Garbaccio R., Leander K., Walsh E., Howell B., Sepp-Lorenzino L., Riley S. (2014). Novel Endosomolytic Poly(amido amine) Polymer Conjugates for Systemic Delivery of siRNA to Hepatocytes in Rodents and Nonhuman Primates. Bioconjug. Chem..

[121] Duggan S.T., Keating G.M. (2011). Pegylated Liposomal Doxorubicin. Drugs.

[122] Ginn S.L., Amaya A.K., Alexander I.E., Edelstein M., Abedi M.R. (2018). Gene therapy clinical trials worldwide to 2017: An update. J. Gene Med..

[123] Torchilin V.P. (2005). Recent advances with liposomes as pharmaceutical carriers. Nat. Rev. Drug Discov..

[124] Hennink W.E., van Nostrum C.F. (2012). Novel crosslinking methods to design hydrogels. Adv. Drug Deliv. Rev..

[125] Ran F.A., Hsu P.D., Wright J., Agarwala V., Scott D.A., Zhang F. (2013). Genome engineering using the CRISPR-Cas9 system. Nat. Protoc..

[126] Rui Y., Wilson D.R., Green J.J. (2018). Non-Viral Delivery To Enable Genome Editing. Trends Biotechnol..

